# 中国儿童急性早幼粒细胞白血病诊断与治疗指南（2025年版）

**DOI:** 10.3760/cma.j.cn121090-20241206-00541

**Published:** 2025-06

**Authors:** 中国医师协会儿科医师分会儿童血液肿瘤学组 中华医学会儿科分会血液学组 中华医学会儿科分会肿瘤学组

**Keywords:** 白血病，早幼粒细胞，急性, 儿童, 指南, Leukemia, promyelocytic, acute, Pediatric, Guideline

## Abstract

急性早幼粒细胞白血病（APL）是急性髓系白血病的一种特殊亚型，常以严重出血为首发表现，起病凶险。虽然APL的5年无病生存率已达90％以上，但在儿童APL诊断和治疗策略上仍存在诸多问题。为了推动儿童APL诊疗水平的全面提升，我们制定了基于循证医学证据的中国儿童APL诊疗指南。

## Abstract (en)

Acute promyelocytic leukemia（APL）, a distinct subtype of acute myeloid leukemia, often presents with severe bleeding as its initial manifestation, posing a significant diagnostic and therapeutic challenge because of its aggressive onset. Although the 5-year disease-free survival rate for APL now exceeds 90%, substantial hurdles remain in optimizing the diagnostic and treatment protocols for pediatric APL. To improve the overall standard of care and address these challenges, the Chinese diagnosis and treatment guideline for pediatric APL grounded in robust evidence-based medical research was developed.

急性早幼粒细胞白血病（APL）是急性髓系白血病（AML）的一种特殊亚型，占儿童AML的10％左右。常以严重出血为首发表现，起病凶险，可导致早期死亡。虽然近年来采用全反式维甲酸（ATRA）联合砷剂诱导分化治疗，APL的5年无病生存（DFS）率达90％以上，但儿童APL诊断和治疗策略仍存在诸多问题，阻碍了儿童APL诊疗水平的一致性和同质化进步。目前国际上有关APL的指南主要针对成人APL患者，如2019版欧洲白血病网（European LeukemiaNet，ELN）共识[1]和2023版美国国立综合癌症网络（National Comprehensive Cancer Network, NCCN）指南[2]。而国内现有的成人和儿童APL诊疗指南[3]–[4]亟需结合最新研究成果与循证医学证据进行更新与优化。因此，制定基于循证医学证据的中国儿童APL诊疗指南尤为迫切，这将为中国儿童APL的临床诊断和治疗提供决策依据，推动儿童APL诊疗水平的全面提升。

## 制定方法

1. 指南发起机构：由中国医师协会儿科医师分会儿童血液肿瘤学组、中华医学会儿科分会血液学组和肿瘤学组共同发起。启动时间为2022年9月，定稿时间为2024年11月。

2. 指南制定工作组建立：指南工作组包括指导委员会（6人）、专家共识组（26人）、秘书组（证据评价组）（13人）、指南外审组（5人）。专家共识组和证据评价组各包含1名循证医学专家。证据检索由北京儿童医院图书馆完成。所有工作组成员均填写利益冲突声明表，与本指南无利益冲突。

3. 指南注册：已在国际实践指南注册与透明化平台（International Practice Guidelines Registry Platform）注册，注册号：PREPARE-2022CN515。

4. 指南使用者与目标人群：本指南供各等级医院从事儿童APL诊治相关工作的临床医师、卫生决策人员和相关科研工作人员使用。目标人群为1岁以上且18岁以下的PML::RARα阳性和（或）具有t（15;17）（q22;q21）的APL患者。

5. 指南问题遴选、证据检索：基于国内外儿童APL指南及高质量系统评价整理出45个临床问题，再对9家医院的12位临床医师进行访谈后增加4个临床问题。通过2轮德尔菲问卷调研和1轮专家会议，最终遴选出拟解决的12个临床问题。证据评价组按照人群、干预、对照、结局的原则进行检索，涵盖Pubmed、Medline、Cochrane Library、Web of Science、Embase、中国生物医学文献服务系统、万方知识数据服务平台和中国知网数据库，纳入文献类型包括系统评价、Meta分析、随机对照试验、队列研究、病例对照研究、病例系列、指南、专家共识等。

6.证据分级：采用2009版英国牛津大学循证医学中心的证据分级与推荐强度标准对推荐意见的证据水平和推荐强度进行分级（表1）（https://www.cebm.ox.ac.uk/resources/levels-of-evidence/oxford-centre-for-evidence-based-medicine-levels-of-evidence-march-2009）。对于设计良好的单臂临床试验，结合2001版牛津分级，按照与单个队列研究同级归为证据水平2b，推荐强度B。本指南同时纳入成人和儿童APL研究进行证据分级。此外，鉴于临床问题1为公认的定义性问题，本指南不对其进行证据等级和推荐强度分级评估。

**表1:** 2009版牛津大学循证医学中心的证据分级与推荐强度标准

推荐强度	证据水平	定义（以治疗/预防为例）
A	1a	纳入一致性较高的随机对照试验的系统评价
	1b	置信区间较窄的单个随机对照试验
	1c	“全或无”证据（有治疗前，所有患者均死亡；有治疗后，有患者能存活。或有治疗前，部分患者死亡；有治疗后，无患者死亡）
B	2a	纳入一致性较高的队列研究的系统评价
	2b	单个队列研究（或低质量随机对照试验）
	2c	结果研究或生态学研究
	3a	纳入一致性较高的病例对照研究的系统评价
	3b	单个病例对照研究
C	4	病例系列（或低质量队列研究或病例对照研究）
D	5	专家意见

7.推荐意见的形成：证据评价小组基于APL研究现状，形成证据总结和初拟的推荐意见。根据德尔菲调研结果，专家共识率>75％视为达成共识。再基于专家提出的反馈意见进行完善。

8.指南推广与实施：本指南发布后将有计划地在全国组织学术会议进行指南介绍和解读，组织儿科等相关领域医务工作者学习。

9.指南的更新：计划在指南发布后3～5年依据国际指南更新流程进行更新。

## 推荐意见及依据

**临床问题1：对儿童APL具有直接诊断意义的关键指标是什么？**

**【推荐意见】**

**1. 通过染色体核型分析、荧光原位杂交（FISH）和（或）聚合酶链反应（PCR）检出t（15;17）（q22;q21）、PML::RARα融合基因是诊断儿童APL的关键指标。**

**2. 推荐有条件时应进行定量PCR检测，明确PML::RARα亚型，便于治疗后基因微小残留病（MRD）的监测。**

【证据概述】

t（15;17）（q22;q21）、PML::RARα融合基因是儿童APL诊断的关键。染色体核型分析、FISH、PCR检测到t（15;17）（q22;q21）和（或）PML::RARα，无论细胞形态学是否符合AML-M_3_，或骨髓白血病细胞比例是否超过20％，均可确诊APL[5]。2017年日本血液学会、ELN共识均推荐有条件时应进行定性或定量PCR检测，明确PML::RARα亚型，便于治疗后基因MRD的监测[1],[6]。

【推荐说明】

由于少数骨髓形态学为M_3_的患者无PML::RARα融合基因，而有其他类型的RARα易位（变异型APL），如t（11;17）（q23;q21）/PLZF::RARα、t（5;17）（q35;q21）/NPM::RARα和t（11;17）（q13;q21）/NuMA::RARα，可能对ATRA和三氧化二砷（ATO）治疗效果欠佳[7]。因此，建议同时应用染色体核型分析及FISH、PCR方法检测明确诊断，以确定治疗方案。

**临床问题2：初治APL的危险度分层因素？**

**【推荐意见】**

**1. 推荐将初诊外周血WBC≥10×10^9^/L作为初治APL的预后不良因素（证据等级2b，推荐强度B）。**

**2. 推荐将巩固治疗后分子生物学持续不缓解（PML::RARα阳性或>10^−4^）作为初诊APL预后不良的高危因素（证据等级2b，推荐强度B）。**

**3. 在包含砷剂的APL治疗方案中，不推荐将FLT3-ITD及其他遗传学异常作为不良预后的高危因素（证据等级2b，推荐强度B）。**

【证据概述】

初诊外周血WBC对于初治APL的预后意义共纳入9个原始研究（包含3个队列研究，4个病例对照研究，2个病例系列，共1 765例）[8]–[16]：初诊时WBC>10×10^9^/L的儿童及青少年患者的总生存（OS）率（*OR*＝0.30，95％*CI*：0.16～0.54，*P*＝0.01）和无事件生存（EFS）率（*OR*＝0.04，95％*CI*：0.03～0.07，*P*<0.01）较低，主要与分化综合征（differentiation syndrome, DS）和早期死亡风险增加相关。由于儿童APL研究中多以WBC≥10×10^9^/L划分为高危[17]–[18]，故本指南推荐将初诊WBC≥10×10^9^/L作为初治APL的预后不良因素。

PML::RARα转录本监测对于初治APL的预后意义共纳入3个原始研究（包含2个队列研究和1个病例对照研究）：PML::RARα转录本是无复发生存（RFS）最有力的预测因素（*HR*＝17.87，95％ *CI*：6.88～46.41, *P*＝0.0001）[19]。巩固治疗后PML::RARα阳性的患者与阴性患者相比，复发风险明显增加（57％对27％，*P*＝0.006）[20]。两次及以上的PML::RARα阴性结果与长期缓解相关[21]。

FLT3-ITD及其他遗传学异常对于初治APL的预后意义共纳入10个原始研究（包含4个队列研究和6个病例对照研究）和1个专家共识：①FLT3-ITD突变与WBC增加及早期死亡相关，在以化疗为基础的治疗方案中是主要的不良预后危险因素[22]–[25]。但在包含砷剂的治疗方案中，FLT3-ITD突变并未对预后造成不良影响[14]–[15],[26]–[28]。②其他遗传学异常对于预后的影响：1项研究（34例，单用砷剂作为诱导治疗）报告，多基因突变（*HR*＝1.994，95％*CI*：1.216～3.271，*P*＝0.006）、KRAS（*HR*＝5.136，95％*CI*：1.356～19.455，*P*＝0.016）和（或）GATA2突变（*HR*＝4.070，95％*CI*：1.287～12.877，*P*＝0.017）患者的早期死亡率升高[15]。但英国医学研究理事会研究中，其他遗传学异常如复杂核型等对预后无显著影响[29]。ELN专家共识建议在诊断时无需常规检测FLT3-ITD及其他遗传学异常[1]。

【推荐说明】

在既往以ATRA和化疗为主的治疗方案中，FLT3-ITD突变患者初诊时常伴高WBC，早期死亡风险高，OS率较低[22],[24],[30]–[31]，但在接受包含砷剂治疗的患者中没有得到证实。其他遗传学异常如多基因突变、复杂核型对于APL的预后意义目前尚无统一定论。

**临床问题3：疑诊APL时的处理？**

**【推荐意见】**

**疑诊APL时，即使t（15;17）（q22;q21）和（或）PML::RARα结果尚未回报时，也应尽快开始ATRA（证据等级2b，推荐强度B）和砷剂（证据等级5，推荐强度D）治疗。**

【证据概要】

指南证据工作组进行了定性研究，共纳入4项前瞻性研究，2项回顾性研究：一项儿童及成人APL回顾性队列研究显示，WBC>10×10^9^/L患者中，ATRA延迟应用（疑诊APL 3～4 d时开始应用）较疑诊0～2 d开始应用早期死亡率明显增加（80％对20％，*P*＝0.01）[32]。多项研究表明柔红霉素单药治疗APL的时代，早期死亡率可高达25％[33]，随着ATRA[34]及ATO[35]应用于一线治疗，ATRA联合砷剂治疗APL患者的早期死亡率下降至0.6％（1/154）～3％（5/186）[17]–[18]。砷剂在儿童APL治疗中的有效性和安全性已得到证实，ATRA联合砷剂治疗使得APL的早期死亡率进一步下降，因此中国儿童白血病协作组（China Children Leukemia Group，CCLG）-APL协作组建议对于疑诊APL的患者，在尽早开始ATRA治疗的同时也应尽快开始砷剂的治疗。

【推荐说明】

APL早期死亡的最主要原因是重要脏器出血，尤其是颅内出血。疑诊APL时应立即开始ATRA和砷剂治疗，在细胞遗传学和分子生物学不支持APL诊断后停用。

**临床问题4：对于初诊WBC<10×10^9^/L的APL的一线治疗方案？**

**【推荐意见】**

**1. 对于初诊WBC<10×10^9^/L的APL首选ATRA联合砷剂作为一线治疗方案（证据等级1a，推荐强度A）。详见表2。**

**表2:** 急性早幼粒细胞白血病（APL）治疗方案推荐

药物	诱导治疗	巩固治疗	维持治疗
ATRA	15～25 mg·m^−2^·d^−1^，分1～2次口服，第1～28天	15～25 mg·m^−2^·d^−1^，分1～2次口服，第1～14天	15～25 mg·m^−2^·d^−1^，分1～2次口服，用1周停1周，依次循环，每8周1个疗程，共4个疗程
砷剂（选其一）			
ATO	0.15 mg·kg^−1^·d^−1^，最大量10 mg/d，静滴大于6 h，第1～28天	0.15 mg·kg^−1^·d^−1^，最大量10 mg/d，静滴大于6 h，第1～14天	0.15 mg·kg^−1^·d^−1^，最大量10 mg/d，静滴大于6 h，用2周停2周，每8周1个疗程，共4个疗程
RIF	50～60 mg·kg^−1^·d^−1^，分2～3次口服，可根据血砷浓度调整剂量，最大量135 mg·kg^−1^·d^−1^，第1～28天	50～60 mg·kg^−1^·d^−1^，分2～3次口服，可根据血砷浓度调整剂量，最大量135 mg·kg^−1^·d^−1^，第1～14天	50～60 mg·kg^−1^·d^−1^，分2～3次口服，可根据血砷浓度调整剂量，最大量135 mg·kg^−1^·d^−1^，第1～28天，用2周停2周，每8周1个疗程，共4个疗程
蒽环类药物（高危选其一^a^）			
伊达比星	10 mg·m^−2^·d^−1^，共2～3次	10 mg·m^−2^·d^−1^，共2～3次	
柔红霉素	40 mg·m^−2^·d^−1^，共2～3次	40 mg·m^−2^·d^−1^，共2～3次	

**注** 治疗期间需根据患儿治疗反应调整用药。ATO：三氧化二砷；RIF：四硫化四砷。^a^对于初诊APL，诱导治疗阶段若白细胞控制较好，可减少蒽环类药物的使用（包括减少蒽环类的剂量或次数），甚至不用蒽环类药物

**2. 口服复方黄黛片（Realgar-Indigo naturalis formula，RIF，活性成分为四硫化四砷）可应用于儿童APL的治疗（证据等级1a，推荐强度A），建议口服RIF时监测砷浓度（证据等级5，推荐强度D）。**

【证据概要】

首选ATRA联合砷剂（ATO/RIF）作为一线治疗方案的评价共纳入2个Meta分析、2个单臂临床试验、1个RCT研究：1个Meta分析[36]（6个RCT、2个CCT，共480例）显示与ATRA单药相比，ATRA + ATO联合治疗显著改善完全缓解（CR）率（*RR*＝1.09, 95％*CI*：1.03～1.16，*P*＝0.004），降低早期死亡率（*RR*＝0.42, 95％*CI*：0.20～0.9，*P*＝0.03）和复发率（*RR*＝0.17, 95％*CI*：0.07～0.42，*P*<0.0001）。另1个Meta分析[37]（2个RCT、1个前瞻性研究，共585例）显示与接受ATRA+化疗的患者相比，ATRA+ATO患者的EFS率（*HR*＝0.38, 95％*CI*：0.22～0.67, *P*＝0.009）、OS率（*HR*＝0.44, 95％ *CI*：0.24～0.82, *P*＝0.009）显著提高。中国的1个儿童前瞻性单臂多中心研究（CCLG-APL2016）（186例）[17]显示WBC<10×10^9^/L的APL患儿采用“ATRA+ATO/RIF”完全去化疗的2年OS率为99％，EFS率为97％。美国COG的1个儿童前瞻性单臂多中心研究[18]中98例WBC<10×10^9^/L患者采用ATRA+ATO完全去化疗治疗后2年OS率为99％，2年EFS率为98％。中国的1个儿童RCT研究[38]结果初步显示在WBC≤10×10^9^/L的患者中，诱导期采用ATRA+砷剂+1剂蒽环类药物（139例）治疗的患儿在出凝血事件（*P*<0.001）、DS（*P*<0.001）方面优于ATRA+砷剂去化疗组（27例）。

口服砷剂应用于APL治疗的评价共纳入1个Meta分析和1个RCT研究：1个Meta分析[39]（4个RCT，482例）显示与静脉注射砷剂ATO组相比，口服砷剂RIF组更易获得CR，但差异无统计学意义（pooled *OR*＝4.59, 95％ *CI*：0.74～28.57, *P*＝0.10, *I*^2^＝0％）。其他疗效指标，包括30 d死亡率（pooled *OR*＝0.22, 95％*CI*：0.04～1.36, *P*＝0.10, *I*^2^＝0％）、OS（pooled *OR*＝5.48, 95％*CI*：0.91～32.86, *P*＝0.06, *I*^2^＝0％）和EFS（pooled *OR*＝2.87, 95％*CI*：0.73～11.33, *P*＝0.13, *I*^2^＝0％），提示口服砷剂RIF组疗效可能更好，差异尚未达统计学意义。华南儿童白血病协作组（South China Children Leukemia Group，SCCLG）-APL多中心RCT研究[40]（176例）显示，尽管口服砷剂RIF（91例）与静脉注射砷剂ATO（85例）的5年EFS率差异无统计学意义，但口服砷剂组平均住院时间更短（WBC≤10×10^9^/L且PLT≥40×10^9^/L，39 d对64 d，*P*＝0.000；WBC≤10×10^9^/L且PLT<40×10^9^/L，34 d对65 d，*P*＝0.000；WBC>10×10^9^/L，44.5 d对68 d，*P*＝0.048），感染风险降低（26.7％对42.4％，*P*＝0.029）。

【推荐说明】

本指南治疗方案的推荐仍以初诊WBC作为划分依据，但在ATRA联合砷剂治疗APL的时代，目前使用的APL危险度的分层体系有待改善。上海交通大学附属瑞金医院对348例成人APL进行全外显子测序、全基因组测序和RNA测序发现，既往仅基于初诊WBC和（或）PLT的危险度分层已经不适用于ATRA联合砷剂治疗APL的时代，提出了基于NRAS突变、APL9评分和WBC进行的危险度分层[41]，但仍需前瞻性临床研究来验证。

虽然研究已证实与静脉注射砷剂联合ATRA相比，口服RIF联合ATRA治疗APL至少具有非劣性，但是在儿童APL中使用口服砷剂，需考虑口服药物的生物利用度和患者的依从性问题，建议至少口服砷剂1周待血砷浓度达稳态后检测谷浓度[17],[40]。

**临床问题5：对于初诊WBC≥10×10^9^/L的APL患者的一线治疗方案？**

**【推荐意见】**

**对于初诊WBC≥10×10^9^/L的APL首选ATRA+砷剂（ATO/RIF）+蒽环类药物（详见表2）作为一线治疗方案（证据等级2b，推荐强度B）。**

【证据概要】

共纳入1个队列研究、3个单臂临床试验、1个RCT研究：一项成人队列研究（对RCT的事后分析）显示，WBC≥10×10^9^/L患者（267例）在采用ATRA+ATO+蒽环类药物治疗的7年DFS率高于ATRA+蒽环类药物+阿糖胞苷（93.2％对87.4％，*P*＝0.14），7年CIR低于ATRA+蒽环类药物+阿糖胞苷组（5.1％对9.9％，*P*＝0.17），尽管差异无统计学意义[42]。中国儿童单臂多中心研究（CCLG-APL2016）显示79例高危组APL患者采用ATRA+ATO/RIF+蒽环类药物（2～3剂）诱导、ATRA+蒽环类药物（3剂）巩固、ATRA+ATO/RIF 5个周期维持治疗，2年OS率为95％，2年EFS率为90％[17]。美国COG的1个儿童单臂多中心研究结果显示，56例WBC≥10×10^9^/L的APL患儿采用ATRA+ATO+蒽环类药物（共4剂）诱导治疗、ATRA+ATO进行4个周期巩固治疗，2年OS率为100％，2年EFS率为96.4％[18]。中国SCCLG-APL协作组的1个多中心随机非劣性研究显示，57例高危APL患者采用ATRA+ATO/RIF+蒽环类药物（米托蒽醌3剂）诱导、ATRA+ATO/RIF+阿糖胞苷巩固、ATRA+ATO+甲氨蝶呤+巯嘌呤维持治疗，8年EFS率高达96％以上[40]。一项单臂多中心2期试验报道，在WBC≥10×10^9^/L的患者中巩固治疗阶段采用口服砷剂联合ATRA去化疗治疗，纳入54例APL患者，随访13.8个月，2年OS率为100％，2年DFS率为94％，所有患者在巩固治疗后均获得分子生物学CR[43]，但中位随访时间较短。因此，各大协作组仍推荐将ATRA+砷剂+蒽环类药物作为WBC≥10×10^9^/L APL的一线治疗方案。

【推荐说明】

尽管各大协作组仍推荐将ATRA+砷剂+蒽环类药物作为WBC≥10×10^9^/L APL的一线治疗方案，但一项成人RCT研究（40例）提示在WBC≥10×10^9^/L的患者，与传统ATRA+ATO+化疗组（19例）相比，ATRA+ATO完全去化疗组（21例）2年DFS率和EFS率更高[44]。因此，对于初诊WBC≥10×10^9^/L的患者有望进一步减化疗、去化疗，但尚需大样本研究进一步证实。此外，维奈克拉在复发APL治疗中效果显著[45]，国内将其应用于APL一线治疗的临床试验已开展，如CCLG-APL2024方案（注册号：ChiCTR2400085721），有望进一步优化APL治疗。

**临床问题6：诱导治疗期间，如何进行减积（降细胞）治疗，从而降低早期死亡？**

**【推荐意见】**

**1. 对于初诊或诱导治疗后WBC>5×10^9^/L的患者，首选羟基脲进行减积治疗（证据等级5，推荐强度D）。**

**2. 对于初诊或诱导治疗后WBC>5×10^9^/L的患者，也可选用维奈克拉进行减积治疗（证据等级4，推荐强度C）。**

**3. 对上述药物减积治疗不敏感的患者，可加用高三尖杉酯碱、阿糖胞苷或蒽环类药物（证据等级5，推荐强度D）。**

【证据概述】

一共纳入涉及APL减积治疗的文献5篇，其中4篇均为临床试验中建议的减积治疗，1篇为历史对照研究，结果见表3。

**表3:** 急性早幼粒细胞白血病（APL）减积治疗汇总

研究	WBC	减积药物
Zhang等[46]儿童临床试验	初诊WBC>20×10^9^/L	羟基脲1.0～2.0 g/d，分2～3次口服。若仍继续增多，则给予高三尖杉酯碱
Zheng等[17]儿童临床试验（CCLG-APL2016）	初诊或诱导后WBC≥10×10^9^/L	羟基脲10～40 mg·kg^−1^·d^−1^，分2～3次口服 阿糖胞苷40～100 mg/m^2^，静脉滴注，每日1次或每12 h 1次 或高三尖杉酯碱1 mg/m^2^，静脉滴注，每日1次
Kutny等[18]儿童临床试验（COG AAML1331）	初诊WBC≥10×10^9^/L	羟基脲120 mg·kg^−1^·d^−1^（不超过4 g/d），分4次口服
Huang等[40]儿童临床试验	初诊或诱导治疗期间WBC>10×10^9^/L	羟基脲100 mg·kg^−1^·d^−1^，直到WBC<10×10^9^/L
Qi等[47]儿童APL减积治疗历史对照研究（11例）	初诊WBC>5×10^9^/L	维奈克拉20～50 mg·m^−2^·d^−1^，每日1次 或羟基脲10～40 mg·kg^−1^·d^−1^，分2～3次口服 或阿糖胞苷40～100 mg/m^2^，静脉滴注，每日1次或每12 h 1次 或高三尖杉酯碱1 mg/m^2^，静脉滴注，每日1次 或蒽环类药物（去甲氧柔红霉素10 mg·m^−2^·d^−1^或柔红霉素40 mg·m^−2^·d^−1^，静脉滴注，2～3次），可酌情将剂量减半使用

【推荐说明】

WBC明显升高是APL患者早期死亡的危险因素[48]–[49]，羟基脲为最常用的减积药物，若单用效果欠佳，可联用化疗药物，如阿糖胞苷、蒽环类药物和高三尖杉酯碱。北京儿童医院单中心研究结果显示，采用维奈克拉进行减积治疗的APL患儿的DS发生率更低，是一种更优的选择[47]。因此，本指南推荐首选羟基脲进行减积治疗，对于不敏感的患者，可加用维奈克拉；若WBC下降仍不明显，可加用高三尖杉酯碱、阿糖胞苷或蒽环类药物。APL的减积治疗通常与ATRA和砷剂靶向治疗同时进行，并不影响APL的诱导分化治疗。

**临床问题7：治疗期间和停药后MRD的监测？**

**【推荐意见】**

**1. 推荐通过定量PCR检测骨髓PML::RARα转录本进行MRD监测（证据等级2b，推荐强度B）。**

**2. 建议监测频率：维持治疗前每个疗程结束后监测1次，维持治疗期每3个月左右监测1次（证据等级2b，推荐强度B），停药时监测1次，之后每6个月监测1次，直至停药后2年（证据等级5，推荐强度D）。**

【证据概述】

指南制订工作组共查询到1个前瞻性研究、4个指南及专家共识：对于MRD监测方法及监测频率，各研究及指南推荐较为一致。多推荐应用定量PCR，包括实时荧光定量PCR（RQ-PCR）和逆转录定量PCR（qRT-PCR），每2～3个月进行一次PML::RARα转录本检测，但监测时长不尽相同（表4）。

**表4:** 微小残留病（MRD）监测情况

研究	监测方法	监测频率及时长
Grimwade等[19]前瞻性研究	RQ-PCR	每个疗程后监测1次，巩固治疗后每3个月监测1次，持续监测到巩固治疗后2年，最长可达3年
2018年中国APL诊疗指南[3]	qPCR	治疗期间每2～3个月监测1次，持续监测2年
2022年中国儿童APL诊疗指南[4]	qRT-PCR	诱导治疗结束不一定需要监测MRD，但巩固治疗结束必须监测MRD（骨髓标本），之后每3～6个月复查（外周血或骨髓标本），至少到维持治疗结束后24个月
ELN关于MRD监测的专家共识[50]	qRT-PCR	诱导治疗结束时；随后每3个月监测1次，对于初诊WBC>10×10^9^/L的患者持续监测至停药后至少2年，对于初诊WBC≤10×10^9^/L的患者在达到MRD阴性后可终止监测
ELN关于APL管理的专家共识[1]	qRT-PCR/RQ-PCR	对于初诊WBC>10×10^9^/L的患者，巩固治疗后每3个月监测1次，至巩固后3年；对于初诊WBC≤10×10^9^/L的患者，巩固治疗后MRD阴性则不再进行MRD监测；不推荐在临床试验之外进行诱导治疗后的MRD评估

**注** RQ-PCR：实时荧光定量PCR；qPCR：定量PCR；qRT-PCR：逆转录定量PCR

【推荐说明】

监测期间若出现PML::RARα阳性，需在2周内复测，若复查阴性，继续原治疗，若为阳性，按照复发治疗。

**临床问题8：中枢神经系统白血病（central nervous system leukemia，CNSL）的预防？**

**【推荐意见】**

**1. 对于初诊WBC≥10×10^9^/L或中枢神经系统（CNS）出血的APL患者，待弥散性血管内凝血（DIC）控制后建议进行至少4次鞘内注射化疗药物预防CNSL（证据等级2b，推荐强度B）。**

**2. 对于初诊WBC<10×10^9^/L且无颅内出血的患者，待DIC控制后建议进行鞘内注射化疗药物预防CNSL，可酌情减少至2～3次（证据等级5，推荐强度D）。**

【证据概要】

在ATRA联合化疗时代，一项成人及儿童队列研究表明，806例APL患者中10例髓外复发的患者中9例复发涉及CNS，APL髓外复发在WBC>10×10^9^/L的患者中更常见（*P*＝0.0014），且预后较差（*P*＝0.04）[51]；另一项739例成人APL患者的队列研究，未进行鞘内注射化疗药物预防CNSL，诱导治疗期间CNS出血的发生（5年累积发生率18.7％，*P*＝0.006）是CNS复发的独立危险因素[52]。

在ATRA联合砷剂时代，AAML1331研究中[18]，仅对非创伤性脑脊液中存在白血病细胞或CNS出血的患者进行了三联鞘内注射，结果显示154例患者中3例复发，其中仅1例WBC<10×10^9^/L复发伴CNS2；国内关于儿童APL研究，如CCLG-APL2016研究[17]、SCCLG-APL[53]，均对所有APL患者进行二联鞘内注射（阿糖胞苷+地塞米松）预防CNSL。在CCLG-APL2016研究中（193例），仅1例患者复发时伴有CNSL；在SCCLG-APL研究中（82例），中位随访时间3.0年无CNS复发患者。

【推荐说明】

APL患者CNSL的发生率很低，而诱导治疗期间出血风险高，2019年ELN专家共识[54]建议在诱导治疗期间应避免腰椎穿刺等有创性操作，CNSL的预防应推迟至血液学缓解后。目前国内推荐对于所有APL患者均进行椎管内注射化疗药物预防CNSL，本指南也推荐对所有患者均进行鞘内注射化疗药物预防CNSL，但需待DIC控制后进行。对于初诊APL合并有颅内出血的患者应根据出血部位、生命体征、影像学评估结果，考虑鞘内注射化疗药物的时机。

**临床问题9：儿童复发APL患者的预后不良因素？**

**【推荐意见】**

**儿童复发APL患者的预后不良因素包括：①复发时间较早：CR_1_<18个月（证据等级4，推荐强度C）；②复发后骨髓造血干细胞移植（HSCT）前未达到CR（证据等级4，推荐强度C）；③初诊时已使用ATO治疗（证据等级2b，推荐强度B）。**

【证据概要】

指南工作组共纳入5项回顾性队列研究，报告了复发APL患者预后不佳的3类危险因素：①复发时间较早（3个研究，共473例）：在一项儿童研究中（24例），从诊断到复发<18个月是复发APL预后不良的危险因素（OS：*HR*＝13.15，95％*CI*：2.65～65.11，*P*＝0.002；EFS：*HR*＝8.94，95％*CI*：2.33～34.36，*P*＝0.001）[55]。在一项儿童及成人研究中（155例），CR_1_≥1.5年是复发APL预后较好的保护因素（OS：*HR*＝0.410，95％*CI*：0.183～0.919，*P*＝0.03；EFS：*HR*＝0.401，95％*CI*：0.210～0.764，*P*＝0.006）[56]；在一项成人研究中（294例）CR_1_<12个月是复发APL预后不良的危险因素（OS：*HR*＝1.56，95％*CI*：1.07～2.26，*P*＝0.02）[57]。②移植前未达到CR是复发APL预后不良的危险因素（1个儿童研究，55例）：移植前未达到CR的患者与复发后达到CR_2_+CR_3_的患者相比，5年OS率［46％（95％ *CI*：17％～71％）对81％（95％*CI*：66％～90％），*P*＝0.013］和DFS率［46％（95％*CI*：17％～71％）对76％（95％*CI*：61％～87％），*P*<0.001］均显著偏低[58]。③初诊时已使用ATO治疗（1个青少年及成人研究，64例）：初始治疗中已应用过ATO但仍复发的APL患者，与未应用过ATO而复发的患者相比OS率（*HR*＝2.56，95％*CI*：1.01～6.52，*P*＝0.048）和RFS率（*HR*＝4.74，95％*CI*：1.99～11.22，*P*<0.0005）更低[59]。

【推荐说明】

较早复发、移植前未达到CR、初诊时已使用ATO治疗与儿童复发APL预后不佳相关。因此，对具有危险因素的APL复发患者需要更积极的治疗。但由于目前儿童APL复发研究较少，其预后不良因素如遗传学异常等需待进一步探索。

**临床问题10：首次复发APL的治疗方案？**

**【推荐意见】**

**1. 建议进行APL细胞ATRA及砷剂耐药变异位点检测（证据等级2b，推荐强度B）。**

**2. 复发后治疗：**

**（1）如初诊时采用ATRA+化疗，建议复发时应用ATRA+砷剂再诱导治疗（证据等级2b，推荐强度B）；**

**（2）如初诊时采用ATRA+砷剂治疗，建议复发后进行如下治疗：**

**①建议进行ATRA+砷剂+维奈克拉+化疗再诱导治疗（证据等级4，推荐强度C）；如存在ATRA耐药变异位点，可去除ATRA，如存在砷剂耐药变异位点，可考虑去除砷剂（证据等级4，推荐强度C）。**

**②如经过ATRA+砷剂+化疗+维奈克拉再诱导治疗后达到分子生物学CR，可结合前期治疗反应、儿童生存质量、儿童及监护人意见综合评估，选择进行异基因HSCT或含有维奈克拉的维持治疗（证据等级5，推荐强度D）；如经过ATRA+砷剂+化疗+维奈克拉治疗后仍然未能达到分子生物学CR，建议行异基因HSCT（证据等级2b，推荐强度B）。**

**3. 对于APL复发伴CNSL的患者，建议在上述复发后治疗的基础上增加鞘内注射次数，可考虑额外应用脑及脊髓放疗或大剂量阿糖胞苷治疗（证据等级5，推荐强度D）。**

【证据概要】

1. 关于复发时进行APL细胞ATRA和砷剂耐药突变检测：纳入2项队列研究、1篇病例系列及2篇成人病例报告，因治疗方式存在异质性故未合并。一项成人复发APL前瞻性队列研究（35例）中（初诊时曾应用砷剂），复发时存在砷剂耐药的患者死亡率更高（84.6％对22.7％，*χ*^2^＝12.612，*P*<0.001）；含有PML基因变异的患者死亡率更高（88.9％对30.8％，*P*＝0.005）；4例同时存在PML和RARα基因变异的患者均未能缓解[60]。一项成人复发APL回顾性队列研究（30例）显示PML的变异与ATO耐药（*P*<0.0001）、复发次数（*P*＝0.001）、早期复发（*P*＝0.013）相关[61]。一项成人病例系列研究（9例）中，复发后根据APL细胞耐药变异检测结果调整治疗，88.9％达到CR，11.1％达到部分缓解[45]。1篇成人病例报告显示初诊时ATRA+ATO治疗的患者，复发后同时存在PML A216V变异和RARα R394W变异，经过ATRA+ATO+化疗治疗未缓解[62]。1篇成人病例报告显示经过ATRA+化疗、ATRA+ATO及异基因HSCT的患者二次复发时检测出PML基因A216V变异[63]。

2. 复发治疗：对于初始治疗未应用ATO，复发后应用ATO再诱导治疗达到分子生物学缓解的患者，共纳入2项回顾性队列研究：一项儿童研究（51例）结果显示初始治疗采用ATRA+化疗，复发时应用ATRA+ATO（18例）的患者比应用ATRA+化疗（33例）的患者10年OS率［94.4％（95％*CI*：84.4％～100％）对72.1％（95％*CI*：58.2％～89.4％），*P*＝0.087］和EFS率［77.8％（95％*CI*：60.8％～99.6％）对62.9％（95％*CI*：48.2％～82.0％），*P*＝0.390］有增高趋势，但未达统计学差异[64]。一项成人队列研究（198例）显示复发前未应用ATO的患者，复发后应用含有砷剂方案再诱导的患者移植后4年RFS率（88.2％对75.0％，*P*＝0.028）和OS率（92.8％对79.8％，*P*＝0.027）均较高[65]。

对于初始治疗应用ATO，后出现复发的APL患者，共纳入1项单臂临床试验、1项回顾性队列研究、1项病例报告、1项探索性临床研究。一项成人单臂临床试验中，24例复发APL患者（初诊应用ATO，未应用ATRA），复发后应用ATO再诱导治疗，19例再次达到缓解[66]。一项成人回顾性队列研究（25例）中，初次复发时，有16例仍然应用ATRA+ATO/四硫化四砷（ATS）再诱导，其中有10例（62.5％）再次达到CR，4例未缓解，2例发生治疗相关性死亡；其余患者复发时应用化疗或吉妥珠单抗奥佐米星，诱导治疗后存活的患者中总体二次复发率高达78.9％[67]。一项成人病例报告显示，1例初始治疗应用ATRA+ATO+化疗的APL成人患者在复发时发现RARα-LBD区域基因突变，再次应用ATO+ATRA效果不佳，应用维奈克拉单药达到CR，后因经济原因未接受移植，巩固治疗4个月后二次复发[68]。一项探索性临床研究纳入5例初始治疗含ATO或RIF的成人复发APL患者，复发后应用V-CAG方案（维奈克拉+阿糖胞苷+阿克拉霉素+G-CSF）治疗后4例达到CR，1例达到部分缓解并序贯HSCT，之后5例均维持CR[45]。

在自体HSCT与异基因HSCT的选择方面，纳入了3项回顾性队列研究和2项前瞻性队列研究（表5）：对于移植前MRD阳性的患者，建议行异基因HSCT。

**表5:** 复发急性早幼粒细胞白血病（APL）造血干细胞移植（HSCT）治疗队列研究汇总

作者	年份	研究类型	研究对象	主要结果
Testi等[64]	2021	回顾性队列研究	儿童（51例）	自体HSCT与异基因HSCT的儿童（移植前MRD均阴性）OS率（87.5％对76.0％）和EFS率（72.9％对70.6％）差异均无统计学意义
Yamamoto等[58]	2020	回顾性队列研究	儿童（95例）	CR_2_阶段（初次复发后达到CR）接受移植的患者中，自体HSCT和异基因HSCT的5年OS率（85％对78％，*P*＝0.648）和CIR（9％对11％，*P*＝0.828）差异均无统计学意义
Dvorak等[69]	2008	回顾性队列研究	儿童（32例）	自体HSCT和异基因HSCT的5年OS率［82％（51％～96％）对76％（55％～90％），*P*＝1.00］和EFS率［73％（95％*CI* 43％～91％）对71％（95％*CI* 50％～86％），*P*＝0.81］相近；自体HSCT复发率偏高（27％对9.5％，*P*＝0.19），但差异无统计学意义
Meloni等[70]	1997	前瞻性队列研究	儿童及成人（15例）	自体HSCT患者中，移植前MRD阳性的患者复发率较高（100％对12.5％，*P*＝0.001）
Lengfelder等[56]	2015	队列研究（包含前瞻及回顾部分）	成人（155例）	异基因HSCT组与自体HSCT组的3年OS率（79％对77％，*P*＝0.81）和CIR（39％对37％，*P*＝0.80）相近，但移植前MRD阳性的患者多采用异基因HSCT

**注** MRD：微小残留病；OS：总生存；EFS：无事件生存；CR：完全缓解；CIR：累积复发率

3. APL复发伴CNSL的治疗：纳入1项病例系列研究、1项队列研究和1项病例报告。一项儿童及成人病例系列研究显示，CNS复发的儿童及成人APL患者（17例），应用ATO+甘露醇治疗后，脑脊液砷浓度约为血砷浓度的99.7％，5年OS率为82.4％，EFS率为52.9％[71]。一项儿童及成人前瞻性队列研究中，CNS复发的APL患者应用不含ATO的系统治疗，联合三联鞘内注射，中位生存时间仍明显低于仅骨髓复发的患者[51]；一项成人病例报告显示，脑脊液中的砷浓度与血浆砷浓度相关性好[72]，提示砷剂能透过血脑屏障。

对于合并CNSL的APL患者的局部治疗，COG的儿童APL研究中有复发APL患者的脑脊液转阴后再进行5～6次三联鞘内注射（甲氨蝶呤+氢化可的松+阿糖胞苷）的报道[18]。一项成人探索性研究（一线治疗未用ATO）在CNS复发后（9例）首选ATO±ATRA治疗，并行多次三联鞘内注射，患者均达到分子生物学缓解，缓解后分别应用全脑放射、移植、大剂量阿糖胞苷[56]。

【推荐说明】

对于初治时含或不含砷剂的APL患者，复发后均推荐采用以砷剂为基础的治疗方案，这与2016年国际专家小组[73]、2020年欧洲肿瘤协会[74]及2018年日本血液学会[75]观点一致。由于初诊已应用砷剂的患者复发后单用砷剂二次复发率较高，建议在复发后ATRA+ATO+化疗基础上联用维奈克拉。维奈克拉为一种口服的小分子BCL-2抑制剂[76]，在儿童AML中已证实其安全性[77]–[79]和有效性[80]。基于以上证据，建议在复发后ATRA+ATO+化疗基础上联用维奈克拉，如治疗后达到分子生物学CR，可结合前期治疗反应、儿童生存质量、儿童及监护人意见综合评估，选择含有维奈克拉的维持治疗或进行异基因HSCT；如经过ATRA+砷剂+化疗+维奈克拉治疗后仍然未达分子生物学CR，建议行异基因HSCT。国外亦有关于吉妥珠单抗奥佐米星单药[81]或联合ATRA及ATO[82]治疗成人复发APL的单臂临床试验的报道。复发APL患儿可酌情参加国内开展的临床试验，如ChiCTR2400082058、ChiCTR2300074053等。

**临床问题11：在APL诱导治疗期间，凝血异常如何处理？**

**【推荐意见】**

**1. 一旦怀疑APL，应尽早给予ATRA（证据等级2b，推荐强度B）和砷剂（证据等级5，推荐强度D）治疗。**

**2. 通过输注单采血小板尽可能维持PLT>30×10^9^/L；如有活动性出血或WBC>10×10^9^/L，则尽可能维持PLT>50×10^9^/L（证据等级4，推荐强度D）。**

**3. 新鲜冰冻血浆、冷沉淀、纤维蛋白原等输注尽可能维持纤维蛋白原>1.5 g/L、INR<1.5～2.0，维持PT及APTT在正常范围内（证据等级2b，推荐强度C）。**

【证据概述】

指南证据工作组进行了定性研究，共纳入3篇与APL诱导治疗期间凝血异常处理相关的回顾性研究。一项儿童及成人APL研究显示，在45例高危APL患者（WBC>10×10^9^/L）中，ATRA延迟至疑诊后3～4 d开始应用比疑诊APL 0～2 d开始应用早期死亡率更高（80％对20％，*P*＝0.01）[32]。一项成人APL病例对照研究（67例）显示，纤维蛋白原水平≤1.5 g/L（*P*＝0.025）、APTT延长（*P*＝0.021）的APL患者早期死亡率更高[48]。一项27例成人APL合并颅内出血患者的病例对照研究中，出现颅内出血时的PLT和纤维蛋白原的中位水平分别为22×10^9^/L和1.24 g/L，其中早期死亡患者与存活患者相比，PT更长［（18.0±6.03）s对（14.7±1.93）s，*P*＝0.029］，D-二聚体水平更高［（22.4±13.55）mg/L对（10.8±10.63）mg/L，*P*＝0.029］[49]。我国2018年APL诊疗指南[3]及ELN2019年APL共识[1]建议一旦怀疑APL，应立即给予ATRA治疗，通过输注新鲜冰冻血浆、纤维蛋白原和（或）冷沉淀、血小板等成分来维持PLT在（30～50）×10^9^/L以上、纤维蛋白原在1.5 g/L以上、INR<1.5～2.0。在诱导治疗期间应继续支持治疗，直到凝血障碍的临床体征和实验室指标恢复正常。砷剂在儿童APL治疗中的有效性和安全性已得到证实[17]–[18]，ATRA联合砷剂治疗使得APL的早期死亡率进一步下降，因此，指南专家组建议在尽早开始ATRA治疗的同时也应尽快开始砷剂的治疗。

【推荐说明】

关于APL患者凝血异常的治疗，一些抗凝、抗纤溶的药物如肝素[83]、人可溶性重组血栓调节蛋白[84]–[85]、氨甲环酸和氨基己酸[86]–[87]也被用于临床，但存在争议，可能会使出血和血栓形成风险增加，故本指南暂不推荐使用。

**临床问题12：DS的危险因素、预防及治疗？**

**【推荐意见】**

**1. 治疗前WBC>5×10^9^/L、肾功能不全和体重指数升高为APL患者发生DS的危险因素（证据等级3b，推荐强度B）。**

**2. 使用糖皮质激素预防DS的益处尚不明确，对于WBC>10×10^9^/L或WBC增长迅速的患者可酌情预防性应用（证据等级5，推荐强度D）。**

**3. 一旦怀疑DS，立即予地塞米松10 mg·m^−2^·次^−1^（单次最大量为10 mg），每日1次，必要时可每12 h 1次，症状体征好转后减停，一般不超过2周（证据等级4，推荐强度C）。根据患者DS严重程度，酌情停用分化剂（ATRA和/或砷剂）（证据等级5，推荐强度D）。**

【证据概要】

共纳入DS危险因素的文献2篇，均为病例对照研究，一项研究（739例）表明治疗前WBC>5×10^9^/L（*P*＝0.021）、肌酐水平升高（*P*＝0.004）与发生严重DS的风险增加相关[13]。在Breccia等[88]的研究中（144例），体重指数升高是发生DS的独立危险因素（95％*CI*：1.5～34.95, *P*＝0.014）。

共纳入预防DS的文献5篇，4篇为临床试验中应用皮质类固醇预防DS的描述性分析，1篇为历史对照研究。共纳入DS治疗相关文献6篇，1篇为历史对照研究，4篇为临床试验中应用皮质类固醇治疗DS的描述性分析，1篇为病例系列研究。结果见表6。

**表6:** 分化综合征（DS）预防及治疗汇总表

研究	预防DS	治疗DS	DS相关预后
CCLG-APL 2016儿童多中心研究（186例）[17]	治疗前WBC≥10×10^9^/L或诱导后WBC迅速上升，地塞米松10 mg·m^−2^·d^−1^，最大量10 mg/d，分1～2次，WBC<10×10^9^/L后减停	地塞米松治疗，症状好转后减停	41％出现DS，无DS相关死亡
COG AAML1331儿童研究（154例）[18]	治疗前或治疗后WBC≥10×10^9^/L，地塞米松2.5 mg/m^2^，每日2次，直至WBC<10×10^9^/L	地塞米松5.8 mg·m^−2^·次^−1^（最大量10 mg/次），静脉注射，每日2次，持续至少3 d，直至症状消退，此时继续以较低预防剂量地塞米松至第14天	24.5％（24/98例）的标危APL患者及30.4％（17/56例）的高危患者出现DS症状，无DS相关死亡
GIMEMA APL0406成人研究（156例）[89]	泼尼松0.5 mg·kg^−1^·d^−1^直至诱导结束	怀疑DS即予地塞米松10 mg/次，每12 h 1次，静脉注射，直到症状消退	16％～19％出现中重度DS，6％患者出现重度DS，2例患者死于DS
Ravandi等[16]研究	甲基泼尼松龙50 mg/d，连续5 d，然后迅速减停	未提及	15.8％（13/82例）出现DS，无DS相关死亡
英国AML17研究[90]	无预防治疗	地塞米松，但具体用量和疗程不详	21％（38/178例）的低危患者、30％（17/57例）高危APL患者出现DS
Montesinos等[13]研究	西班牙LPA99研究（564例）：所有患者均应用泼尼松0.5 mg·kg^−1^·d^−1^，第1~15天；LPA96研究（175例）：当WBC>5×10^9^/L，予地塞米松10 mg/次，每12 h 1次，使用7 d	地塞米松10 mg/次，每日2次；若DS症状仍进展，停用全反式维甲酸	LPA96方案中，30.3％患者发生DS，16.6％发生严重DS。LPA99方案中23％患者发生DS，11.3％发生严重DS。两组差异有统计学意义
Warrell等[91]成人APL研究	无预防治疗	怀疑DS即予地塞米松10 mg/次，每12 h 1次，连续3 d或更长时间	两年内将DS相关死亡率从33％（3/9）降低到无死亡病例

【推荐说明】

目前暂无对所有APL患者使用皮质类固醇预防DS的支持依据，参考上述研究结果，对于WBC明显增高的患者，可酌情加用地塞米松预防DS，但仍需进一步临床试验确证。
